# Correction: Inhibition of mTOR Alleviates Early Brain Injury After Subarachnoid Hemorrhage Via Relieving Excessive Mitochondrial Fission

**DOI:** 10.1007/s10571-024-01477-2

**Published:** 2024-04-22

**Authors:** Yuchen Li, Pei Wu, Jiaxing Dai, Tongyu Zhang, Ji Bihl, Chunlei Wang, Yao Liu, Huaizhang Shi

**Affiliations:** 1 Department of Neurosurgery, The First Affiliated Hospital of Harbin Medical University, Harbin, 150000 Heilongjiang China; 2 Department of Pharmacology and Toxicology, Boonshoft School of Medicine, Wright State University, Dayton, OH 45435 USA

**Correction to: Cellular and Molecular Neurobiology (2020) 40:629–642** 10.1007/s10571-019-00760-x

The original version of this article unfortunately contained error in Fig. 4.

Specifically, Fig. 4a featured partial duplication of images from the Sham group that were used in the author’s previous research. This overlap occurred because the two studies shared a high degree of correlation, and some experiments were conducted concurrently. To minimize the number of animals used, the authors utilized shared data for the Sham group, which unfortunately resulted in the erroneous images.

**Fig. 4:**
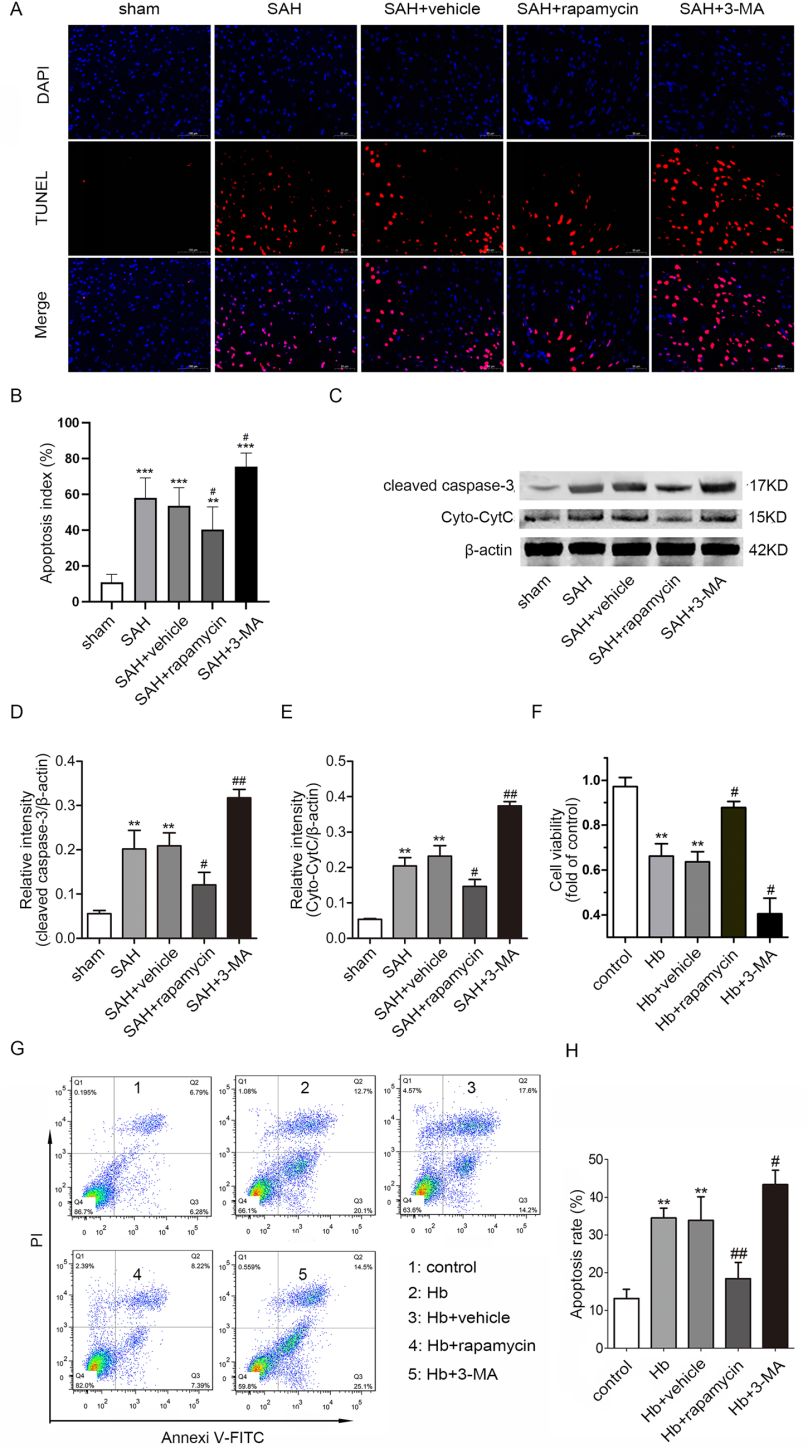
Inhibition of mTOR alleviated neuronal apoptosis after SAH. (**A**) Representative photographs of TUNEL staining in brain sections (scale bar = 50 μm). (**B**) Apoptosis index of the TUNEL staining in each group (*n* = 6). (**C**) Representative images of Cyt C and cleaved caspase-3 bands in rat neurons. (**D**) Quantification of the cleaved caspase-3 expression (*n* = 6). (**E**) Quantification of the Cyt C expression (*n* = 6). The results were normalized to β-actin. (**F**) The cell viability in each group was detected by the MTT assay (*n* = 6). (**G**) The apoptotic rate was assessed by flow cytometry. (**H**) Statistical analysis of the apoptotic rate in each group (*n* = 6, **p < 0.01 and ***p < 0.001 versus sham/control, #*p* < 0.05 and ##*p* < 0.01 versus SAH/Hb)

So, the TUNEL staining experiments again was conducted again and the complete Fig. 4 along with corrected caption and fresh images of Fig. 4a and 4b is given here.

However, the authors assure that this error did not influence the experimental results and conclusions.

The original article has been corrected.

